# A Case of Insanity

**Published:** 1893-06

**Authors:** John H. Fitch

**Affiliations:** New Scotland, New York


### A CASE OF INSANITY.

JOHN H. FITCH, M. D., NEW SCOTLAND, NEW YORK.

Mrs. E. F. D., aged fifty years, married, mother of two children, aged twenty-three and fifteen years. Insane since the spring of 1877, a few months subsequent to the birth of her youngest child. In girlhood had scabies, suppressed with an ointment. Following her confinement she had backache, debility, and a profuse leucorrhœa, which latter complaint was summarily suppressed by a current of electricity applied locally at the hands of a non-graduate. The most violent insane manifestations immediately followed.

December 1st, 1891.—Patient brought home paroled from the Middletown Insane Hospital, where she had been kept fourteen years. Delusion of exalted health and position were present, also the idea that some one had changed her features or stolen her hair, blood, or flesh in her sleep. There was no control over her will, and she would ventilate her delusion or charge the theft above indicated upon any one whom she might meet. A peculiar lustre of the skin of her face was noticeable, resembling polished copper. Ate and slept well and was scrupulously neat in her person. December 19th, Sulphur$^{1m}$, one dose. For the next week or ten days she complained much of the smell of Sulphur on her person and clothing, and hung out all her clothing to air and had a very large laundry.

January 4th, 1892.—Patient was brought to my house, where she remained six months. Although the patient had not been violent, I had a medical student act as body-guard over my family at times of my absence from home. Nothing, however, even occurred to require his services in that capacity.

January 30th.—Her self-esteem having become now a very important feature of the case, together with backache, sensitiveness to cold, irritability of the bladder, etc., Platina.$^{1m}$, one dose.

February 5th.—Patient much less affected by self-esteem, but seemed to have a down-hearted look, and was noticed to be sighing much of the time. A peculiar hacking cough troubling her, a dose or two of Ignatia$^{30}$, was given without much effect when, about February 8th, Calc.$^{1m}$, one dose, was given.

For a month or two patient improved very noticeably. Self-esteem was quite in the background, and quite an amount of control over her will was gained. Friendly chats with members of the family would be held, and neighbors calling would hardly believe that Mrs. D. had spent fourteen years in an insane hospital.

In April visited friends in Albany, her old home, spent a week and returned, traveling alone each way. Toward the close of her visit there I called to see her. Noticed her to be somewhat ill-natured. I gave her Sepia$^{1m}$ in water. She soon after returned to my house. Things went on very well for a month or two, when it was noticed that in her room, especially in the evening, the patient would be talking excitedly, apparently in angry conversation with some one. This had been noticed to a slight extent before at times. It now became a very prominent symptom and would be carried to the extent of a degree of rage, with words very threatening and bordering on profanity. Carbon$^{30}$, in water, and in a few days all was peace.

About July 1st, it not being thought possible by her friends to keep her outside of an asylum, she was taken to the State Insane Hospital at Utica.

I am not sure that the patient can be cured, as her delusions were modified, not abolished, but kept far in the background. I know that a course of carefully selected antipsoric medication, prescribed not on key notes but on totalities diligently compared, greatly relieved her from her delusions and that Cereus Bonplandii wiped out some of the most unpleasant manifestations of her case.

